# Factors Associated With Blood Culture Contamination in Rural Hospitals in Japan: A Cross-Sectional Study

**DOI:** 10.7759/cureus.47987

**Published:** 2023-10-30

**Authors:** Ryuichi Ohta, Chiaki Sano

**Affiliations:** 1 Communiy Care, Unnan City Hospital, Unnan, JPN; 2 Community Medicine Management, Shimane University Faculty of Medicine, Izumo, JPN

**Keywords:** patient care optimization, diagnostic accuracy, staphylococcus epidermidis, escherichia coli, clinical procedures, demographics, bacteremia, rural hospitals, contamination risks, blood culture

## Abstract

Background

Blood culture, a cornerstone diagnostic test, is paramount for identifying bacteremia due to serious infections. However, its accuracy is jeopardized by contamination, often due to inappropriate collection procedures. Resource constraints and a limitation in specialized staff can heighten contamination risks in rural hospitals, underscoring the need to understand the associated demographics and conditions. This study aimed to elucidate the demographics and conditions associated with heightened blood culture contamination risk in rural hospitals to optimize testing practices and improve patient care.

Methods

A single-center, cross-sectional study was conducted in Unnan City Hospital, Unnan, Japan with participants suspected of having bacteremia. Data from the electronic medical records of 455 patients were analyzed using multivariate logistic regression with contamination as the dependent variable.

Results

Of the 455 patients who underwent blood culture testing, 321 and 134 tests were negative and positive for contamination, respectively. Older age and blood obtained from arteries were associated with a reduced risk of contamination (odds ratio (OR)=0.97; p=0.012, and OR=0.17; p=0.00069, respectively). Patients with dependencies exhibited an increase in contamination risk (OR=1.81; p=0.044). Patients admitted for infection demonstrated a reduced likelihood of sample contamination (OR=0.44; p=0.0034). The predominant organisms identified varied, with *Escherichia coli* being more frequent in uncontaminated blood samples and *Staphylococcus epidermidis* in the contaminated samples.

Conclusion

This study reveals a complex relationship between patient demographics, clinical practices, and the risk of contamination. Factors such as age, dependency status, and reason for admission were associated with sample contamination. Enhanced procedural stringency, microbial surveillance, and continuous training could mitigate these risks, particularly in resource-constrained settings. Identifying and understanding the factors influencing blood culture contamination can significantly bolster clinical practice in rural settings. While this study provides foundational insights, future research can deepen our understanding, ensuring the refinement of patient care protocols in similar environments.

## Introduction

Blood culture is a critical diagnostic test that is pivotal for identifying bacteremia across a spectrum of severe infections. This indispensable tool aids healthcare professionals in the diagnosis, management, and treatment of various life-threatening conditions, thereby significantly affecting patient outcomes [1,2]. However, the credibility and accuracy of blood culture results are not immune to the risks associated with contamination [1]. Contamination can arise due to several factors, notably inappropriate procedures or suboptimal conditions at the access location where the blood culture is conducted [3].

In many rural hospitals, there is a shortage of healthcare professionals, a challenge that requires other medical staff who may not specialize in this area to perform blood culture tests [4]. This exigency potentially exacerbates the risk of contamination, as nonspecialist staff may need to be better versed in the meticulous protocols required for accurate blood culture testing [5]. Identifying and understanding the demographics and conditions associated with the heightened probability of blood culture contamination is paramount in this context.

Enhancing the knowledge and awareness of operators conducting blood cultures regarding high-risk demographics and conditions is imperative [6]. This fosters increased vigilance during the testing process and contributes to mitigating contamination risks, ensuring the reliability of blood culture results, and mitigating antimicrobial resistance [7]. In rural settings, where resources may be scarce and specialized staff are limited, such an understanding is vital for optimizing patient care and outcomes.

Against this backdrop, this study aimed to investigate factors associated with t blood culture contamination in rural hospitals, including patient demographics. By identifying these factors, this study aims to contribute to the improvement of blood culture testing practices, increased result accuracy, and subsequent improvement of patient care in rural healthcare settings. Through a nuanced understanding of risk factors, it is anticipated that healthcare workers will be better equipped to avoid risks associated with blood culture contamination, and improve the standard of practice; therefore, optimizing the reliability of this essential diagnostic test in the face of resource constraints and workforce challenges in rural hospitals.

## Materials and methods

This single-center, cross-sectional study aimed to identify patient demographics and other external factors associated with blood culture contamination in rural hospitals. Using data from electronic medical records, multivariate logistic regression was performed with blood culture contamination as the dependent variable. The covariates included age, sex, setting, timing of blood culture, assessment of being care dependent on Japanese long-term health insurance, duration of hospital admission, and diagnosis made.

Setting

In 2022, the total population of Unnan City was 35,738 (17,231 males and 18,507 females), and the percentage of residents aged 65 years and older was 40.27%. There was only one public hospital in the rural area. The rural hospital had 281 beds during the study period, including 155 acute, 48 general, 30 rehabilitation, and 48 chronic. Internal medicine patients are managed by the Department of Family Medicine through collaborative efforts with multiple healthcare professionals [8].

Participants

Study participants were selected from among patients who visited the Unnan City Hospital, Unnan, Japan. Inclusion criteria were: age >18 years, symptoms of infection, and positive blood culture result. Exclusion criteria included the absence of symptoms indicating possible infection, no blood culture, and immunocompromised patients (difficulty in determining whether a positive culture is due to a contaminating organism) [9]. Patient data were collected from the hospital’s electronic medical records between April 2022 and March 2023.

Data collection

Blood culture contamination was used as the dependent variable. Blood culture contamination was defined as cases in which a positive blood culture was judged contamination by the charged physicians, and patients with judged contamination were able to recover without medication or antibiotics among immunocompetent hosts. Risk factors for blood culture contamination were based on previous studies and were evaluated as covariates; data for the covariates were also collected from electronic medical records [1,3]. Covariates included age, sex, setting, timing, being care dependent on Japanese long-term health insurance, blood culture samples, and admission diagnosis. Patients for whom these statements were unavailable were excluded from the study.

Analysis

For continuous variables, the normality of the data was tested before applying statistical tests. Parametric and nonparametric data were analyzed using the Student's t-test and the Mann-Whitney U test, respectively. The chi-squared test was used to analyze categorical data. The following categorical variables were dichotomized for the logistic regression model: sex, setting (outpatient or inpatient), being care dependent on Japanese long-term health insurance (dependent condition or not), blood culture sample location (i.e., artery or vein), and admission diagnosis (infection or not). Multivariate logistic regression analysis was performed to examine the association between blood culture contamination and these factors. Multivariate logistic regression models were constructed using all variables associated with blood culture contamination, and these variables were significant in univariate regression models of blood culture contamination. All data analyses were performed using Easy R, version 1.23 (R Foundation for Statistical Computing, Vienna, Austria). Statistical significance was set at p<0.05.

Ethical consideration

Ethics approval for this study was obtained by the Unnan City Hospital Clinical Ethics Committee (No. 20230019).

## Results

**Figure 1:**
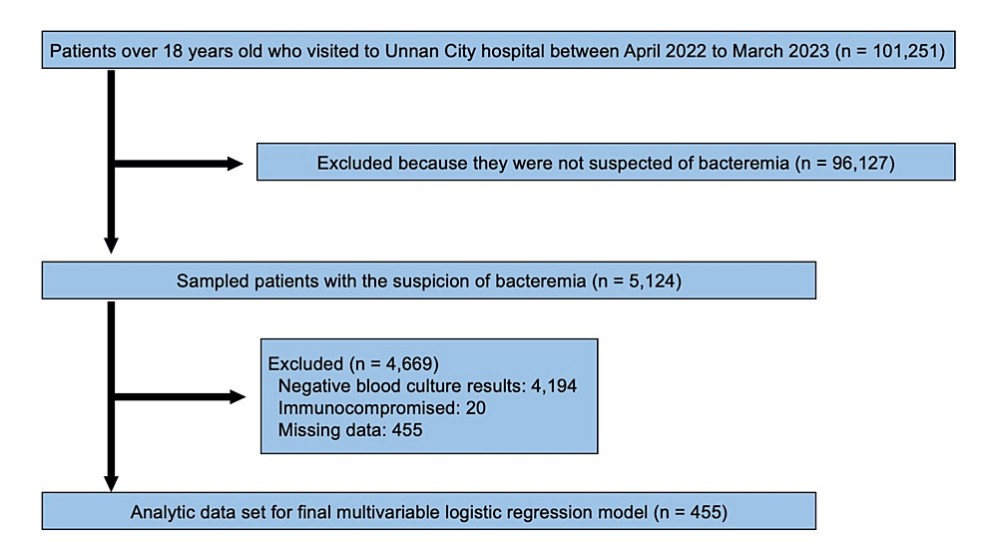
The selection flow of the participants

Figure 1 shows the flowchart of the study participant selection process. Between April 2022 and March 2023, 101,251 patients visited the hospital. Based on medical records, blood culture tests were performed on 5, 124 patients with suspected bacteremia. About 4,669 patients were excluded based on the exclusion criteria. In total, 455 participants were included in this study.

Participant demographics

Of the 455 participants, 321 (70.5%) were negative for contamination, and 134 (29.5%) were found to be contaminated. The average age of all patients was 84.19 years. When assessed by contaminated versus noncontaminated cultures, those in the negative contamination group were slightly older, averaging 85.38 years of age, compared to 81.34 years in the contamination-positive group. This was statistically significant (p<0.001). When broken down by sample retrieval location, 13.4% and 11.9% of samples taken from arteries were negative and positive for contamination, respectively. However, this difference was not statistically significant (p=0.76). A notable difference was observed in reasons for admission. Those who were negative for contamination were significantly more likely to be admitted due to infection, accounting for 40.5% of this group. In contrast, only 23.1% of those with positive contamination results were admitted for the same reason (p<0.001). A significant difference was observed in the time required for the cultures to grow between the two groups. Cultures from the positive contamination group took longer, averaging 6.26 days, compared to just 3.87 days in the negative group (p<0.001) (Table 1).

**Table 1:** The demographic of the participants

		Contamination		
Factor	Total	Negative	Positive	p-value
N	455	321 (70.5)	134 (29.5)	
age, mean (SD)	84.19 (11.29)	85.38 (9.71)	81.34 (14.01)	<0.001
Male sex (%)	232 (51.0)	157 (48.9)	75 (56.0)	0.182
Dependency (%)	118 (25.9)	79 (24.6)	39 (29.1)	0.348
Inpatient (%)	207 (45.5)	145 (45.2)	62 (46.3)	0.837
Admission because of Infection (%)	161 (35.4)	130 (40.5)	31 (23.1)	<0.001
Location for sample: artery (%)	59 (13.0)	43 (13.4)	16 (11.9)	0.76
Timing for blood culture, mean (SD)	15.87 (35.68)	16.63 (38.28)	14.04 (28.55)	0.482
Duration for culture growth (days), mean (SD)	4.57 (2.42)	3.87 (1.59)	6.26 (3.13)	<0.001

Organisms identified in blood cultures

The most frequently identified organism in the negative contamination group was *E. coli,* which accounted for 36.1% of cases. For those with positive contamination, *S. epidermidis *emerged as the predominant contaminating organism, found in 24.6% of cases (Table 2).

**Table 2:** The growing organisms in the blood culture

Contamination			
Negative		Positive	
N (%)	321 (70.5)	N (%)	134 (29.5)
Escherichia coli	116 (36.1)	Staphylococcus epidermidis	49 (36.5)
Staphylococcus aureus MRSA	32 (10.0)	Gram-positive rod	10 (7.5)
Staphylococcus aureus	29 (9.0)	Staphylococcus hominis	10 (7.5)
Klebsiella pneumoniae	26 (8.1)	Corynebacterium sp	9 (6.7)
Escherichia coli ESBL	23 (7.2)	Staphylococcus hominis subsp. hominis	9 (6.7)
Enterococcus faecalis	14 (4.4)	*Streptococcus anginosus *group	4 (3.0)
Klebsiella oxytoca	14 (4.4)	Staphylococcus capitis subspecies capitis	4 (3.0)
Proteus mirabilis	10 (3.1)	Gram-positive cocci	3 (2.2)
Streptococcus dysgalactiae	7 (2.2)	Staphylococcus auricularis	3 (2.2)
Enterobacter cloacae	6 (1.9)	Staphylococcus simulans	3 (2.2)
Pseudomonas aeruginosa	6 (1.9)	Clostridium ramosum	2 (1.5)
Clostridium perfringens	5 (1.6)	Fusobacterium necrophorum	2 (1.5)
Candida parapsilosis	4 (1.2)	Rothia mucilaginosa	2 (1.5)
Morganella morganii	4 (1.2)	Staph capitis subspecies ureolyticus	2 (1.5)
Enterococcus faecium	3 (0.9)	Streptococcus intermedius	2 (1.5)
Serratia marcescens	3 (0.9)	Streptococcus species	2 (1.5)
Yersinia frederiksenii	3 (0.9)	Aerococcus urinae	2 (0.7)
*Aeromonas hydrophila *group	2 (0.6)	Bacillus species	2 (0.7)
*Citrobacter freundii* complex	2 (0.6)	Bacteroides ovatus	2 (0.7)
Enterobacter agglomerans	2 (0.6)	Clostridium innocuum	2 (0.7)
Enterobacter asburiae	2 (0.6)	Gram-negative rod	1 (0.7)
Enterococcus raffinosus	2 (0.6)	Staph hominis subsp. novobiosepticus	1 (0.7)
Proteus vulgaris	2 (0.6)	Staphylococcus capitis subspecies ureoly	1 (0.7)
Raoultella (K.) ornithinolytica	2 (0.6)	Staphylococcus intermedius	1 (0.7)
Enterobacter aerogenes	1 (0.3)	Staphylococcus lugdunensis	1 (0.7)
Enterococcus casseliflavus	1 (0.3)	Staphylococcus sciuri	1 (0.7)
Pseudomonas fluorescens/putida	1 (0.3)	Staphylococcus warneri	1 (0.7)
		Staphylococcus xylosus	1 (0.7)
		Streptococcus sanguis	1 (0.7)

Multivariate logistic regression insights

The effectiveness of the analysis model, determined by the area under the curve, was rated at 0.829, with a 95% confidence interval (CI) ranging from 0.787 to 0.87, indicating the robustness of the findings. Each incremental year of age was associated with a 3% reduction in the odds of infection (odds ratio (OR), 0.97; 95% CI: 0.95-0.99; p=0.012). Participants with dependencies exhibited 81% increased odds of contamination (OR=1.81; 95% CI: 1.02-3.21; p=0.044). Those admitted due to infection had a 56% reduced risk of contamination (OR=0.44; 95% CI: 0.26-0.76; p=0.0034). Drawing samples from arteries was associated with an 83% reduced risk of contamination (OR=0.17; 95% CI: 0.06-0.47; p=0.00069). For every unit increase in the time taken for bacterial growth, there was an 89% increase in contamination odds (OR=1.89; 95% CI: 1.61-2.22; p<0.001) (Table 3).

**Table 3:** The result of the multivariate logistic regression model

Factor	Odds ratio	95% CI	p-value
Age	0.97	0.95-0.99	0.012
Male sex	1.2	0.73-1.98	0.48
Dependency	1.81	1.02-3.21	0.044
Admission due to Infection	0.44	0.26-0.76	0.0034
Location of sample: artery	0.17	0.06-0.47	<0.001
Timing for blood culture	0.99	0.99-1.00	0.09
Duration for bacteria growth	1.89	1.61-2.22	<0.001

## Discussion

Our study yields valuable data on risk factors for blood culture contamination and the associated variables in clinical environments. While this study has considerable implications for practice and policy, several points merit a detailed discussion.

Our finding of a 3% reduction in contamination odds with each year’s increase in age is intriguing. Given their vulnerability, physicians and nurses are more cautious with older individuals and therefore, use more alcohol for cleaning the area where blood is drawn, thereby reducing opportunities for contamination [10]. Their physiological responses, including changes in the skin flora or diminished immune responses, may also play a role [11]. Comparisons with earlier studies yielded mixed outcomes, with some echoing our findings and others demonstrating opposing trends [10,11]. The reasons for these discrepancies merit further in-depth and focused research.

It is concerning and not wholly surprising that participants with dependencies had an 81% increased risk of contamination. Due to their reliance on medical devices or frequent interventions, such patients may inherently face more exposure points, elevating the chances of contamination [12]. This underscores the need for healthcare settings to deploy enhanced care measures and perhaps even specialized teams to manage dependent patients [13].

The markedly prolonged growth in the positive contamination group presents a two-fold concern. First, it might hint at a distinct microbial ecology that could involve slow-growing organisms or organisms that are less commonly encountered in clinical scenarios [14]. Second, it may indicate potential delays in optimal treatments, leading to prolonged culture durations [15]. Both possibilities have direct clinical implications, stressing the need for rapid diagnostic tools and timely therapeutic interventions.

One counterintuitive finding of this study was that those admitted because of infection had a diminished risk of contamination. This could be ascribed to heightened clinical alertness when managing infectious cases, with more stringent adherence to sterilization and procedural protocols [16]. Conversely, the microbial landscape of a patient during infection is dominated by pathogens, making it challenging for contaminants to thrive [17]. Although speculative, this avenue is worth exploring in studies on microbial competition.

Arterial blood culture samples may have a lower contamination rate for the same reason. Medical professionals use meticulous protocols and demonstrate high caution when taking artery samples because of the risk of infection and bleeding [18]. In addition, the invisibility of arteries during the procedure can increase the concentration of blood culture performers compared with venous sampling for blood culture [19]. For practical blood sampling to avoid contamination, access to the arteries can be an option in rural community hospitals.

Our results emphasize the importance of microbial surveillance. There are clear diagnostic and therapeutic messages, with *E. coli* reigning supreme in the negative contamination group and *S. epidermidis *in the positive cohort [20]. First, it underscores the need for hospitals to have an updated antibiogram, given the potential resistance patterns of these dominant organisms [21]. Second, from a diagnostic perspective, frequent encounters with these organisms may necessitate protocol reviews to ensure timely and accurate identification [22-24].

Our study, although offering valuable insights, has certain limitations. First, the research was constrained by its observational nature, which may have limited the establishment of causal relationships. Although substantial, our sample size was drawn from specific settings, potentially affecting the generalizability of our findings to broader populations or varied healthcare environments. We did not account for potential confounders, such as healthcare staff experience or patient comorbidities, which might influence contamination risks. Additionally, categorizing them into negative and positive contamination groups might overlook nuanced differences in microbial ecology. Last, temporal changes in clinical protocols or microbial resistance patterns were not considered, which could have dynamic impacts on the outcomes.

## Conclusions

Our study is a testament to the intricate interplay between patient characteristics, clinical procedures, and the risk of contamination. The pronounced influence of factors such as age, dependency, and reasons for admission on contamination offers clear avenues for clinical intervention and optimization. The microbial insights from this study can guide diagnostic and therapeutic refinement. Although our research provides a solid foundation, it also underscores the need for future investigations to unravel the mechanisms underlying these findings. By doing so, we can aspire to continually enhance the quality of patient care, minimize clinical risks, and foster better outcomes in healthcare settings.
